# Genetic association of ACE2 rs2285666 (C>T) and rs2106809 (A>G) and susceptibility to SARS-CoV-2 infection among the Ghanaian population

**DOI:** 10.3389/fgene.2025.1555515

**Published:** 2025-05-26

**Authors:** Alexander Owusu Boakye, Christian Obirikorang, Anthony Afum-Adjei Awuah, Evans Asamoah Adu, Doris Winter, Eric Ebenezer Boham, Hakim Alani, Sylvester Kofi Newton, Nana Safi Toure Almoustapha, James Deke, Welbeck Odame Dzadey, Louis Adu-Amoah, Sally-Ann Kroduah, Mary Ama Grant, Gracelyn Asare, Amos Amoako-Adusei, Wibke Loag, Jenny Kettenbeil, Yaw Adu Sarkodie, Ebenezer Oduro-Mensah, Alfred Edwin Yawson, Stephen Apanga, Rose Odotei Adjei, Austin Gideon Adobasom-Anane, Eva Lorenz, Aurélia Souares, Oumou Maiga-Ascofaré, Jürgen May, Nicole S. Struck, John Humphery Amuasi

**Affiliations:** ^1^ Kumasi Centre for Collaborative Research in Tropical Medicine (KCCR), Kwame Nkrumah University of Science and Technology, Kumasi, Ghana; ^2^ Department of Molecular Medicine, School of Medicine and Dentistry, College of Health Sciences, Kwame Nkrumah University of Science and Technology (KNUST), Kumasi, Ghana; ^3^ Infectious Disease Epidemiology, Bernhard Nocht Institute for Tropical Medicine, Hamburg, Germany; ^4^ Department of Clinical Microbiology, School of Medicine and Dentistry, College of Health Sciences, Kwame Nkrumah University of Science and Technology (KNUST), Kumasi, Ghana; ^5^ Department of Global and International Health, School of Public Health, College of Health Sciences, Kwame Nkrumah University of Science and Technology (KNUST), Kumasi, Ghana; ^6^ Ga East Hospital, Accra, Ghana; ^7^ Department of Community Health, Medical School, University of Ghana, Accra, Ghana; ^8^ Department of Community Health and Preventive Medicine, School of Medicine, University for Development Studies, Tamale, Ghana; ^9^ German Center for Infection Research (DZIF), Partner Site Hamburg-Borstel-Lübeck-Riems, Hamburg, Germany; ^10^ German Center for Infection Research (DZIF), Partner Site Heidelberg, Heidelberg, Germany; ^11^ Heidelberg Institute of Global Health (HIGH), Heidelberg University Hospital, Heidelberg, Germany; ^12^ Department of Tropical Medicine I, University Medical Centre Hamburg Eppendorf (UKE), Hamburg, Germany

**Keywords:** angiotensin-converting enzyme 2 (ACE2), single nucleotide polymorphism (SNP), SARS-CoV-2 susceptibility, genetic epidemiological study, SARS-CoV-2 infection (COVID-19), candidate gene association study (CGAS)

## Abstract

**Background:**

Severe acute respiratory syndrome coronavirus 2 (SARS-CoV-2), enters human cells using the angiotensin-converting enzyme 2 (ACE-2) receptor. ACE2 single nucleotide polymorphisms (SNPs) can influence susceptibility by affecting viral binding or gene expression. This study investigated the association between ACE2 SNPs, rs2285666 and rs2106809, and the SARS-CoV-2 infection susceptibility in a Ghanaian population.

**Methods:**

Genomic DNA was extracted, using a magnetic bead-based method, from blood samples of a random-subset of 1,334 participants drawn from a two-stage cluster, population-based household cross-sectional SARS-CoV-2 IgG seroprevalence survey. Data collected included, socio-demographic characteristics, medical history, vaccination, and smoking status. Genotyping of the ACE2 SNPs was performed using Allele-Specific Oligonucleotide Polymerase Chain Reaction (ASO-PCR) combined with melting curve analysis. Logistic regression models were utilized to assess the association between the ACE2 SNPs and the susceptibility to SARS-CoV-2 infection

**Results:**

The median age of participants was 33 [Interquartile range (IQR) = 24–46] years. Females accounted for the majority of the sampled population, 64.3%. SARS-CoV-2-IgG seropositivity was (58.4%, 95%CI: 52.6%–64.2%) among the male population and (54.1%, 95%CI: 49.54%–58.61%) in the female population. There were no significant differences in overall allele or genotype frequencies of ACE2 SNPs between SARS-CoV-2 IgG seropositive and seronegative individuals for both females and males. Among females, those with the T allele of ACE2 rs2285666 had a 38% decreased susceptibility to SARS-CoV-2 infection under the dominant [adjusted odds ratio (aOR) = 0.62; 95%CI = 0.45–0.85, P = 0.003] and heterozygous advantage models (aOR = 0.62; 95%CI = 0.45–0.86, P = 0.004), after adjusting for confounders, but not thee recessive model (aOR = 0.41; 95%CI = 0.03–5.22, P = 0.490). No significant association was observed among males. Overall, the ACE2 rs2106809 was not associated with the susceptibility to SARS-CoV-2 infection in both males and females.

**Conclusion:**

This study found no association between ACE2 rs2106809 genetic variant and susceptibility to SARS-CoV-2 infection, whilst the rs2285666 T-allele was associated with a decreased frequency for SARS-CoV-2 infection among Ghanaian females. These findings enhance our understanding of genetic factors influencing SARS-CoV-2 susceptibility, which could help identify at-risk populations and inform more targeted public health interventions in future outbreaks.

## Introduction

The severe acute respiratory syndrome coronavirus 2 (SARS-CoV-2) was identified as the causative agent of the recent global pandemic, coronavirus disease 2019 (COVID-19) (Zhu et al., 2020). Declared as a pandemic in early 2020 (WHO, 2020b), this outbreak significantly disrupted global health systems, societal norms, and economic structures, resulting in widespread morbidity and mortality. As of June 2024, over 700 million confirmed cases and 7.1 million deaths have been recorded globally. Through a combination of scientific innovation, global collaboration, and coordinated public health efforts, the world has made remarkable progress in recovering from the COVID-19 pandemic. The rapid development, approval, and rollout of vaccines have played a critical role in the process. As such, focus has shifted from emergency response to managing COVID-19 as an endemic situation, with the WHO officially announcing it no longer a Public Health Emergency of International Concern (PHEIC) (Statement on the fifteenth meeting of the IHR, 2005). Despite the progress made, it is important to remain vigilant, as the virus still looms as a formidable challenge to global health systems due to its ability to evolve and persist, and also the risk of other emerging infections remains.

The SARS-CoV-2 virus penetrates human cells via attachment of the trimeric SARS-CoV-2 spike (S) protein receptor binding domain (RBD) to the angiotensin-converting enzyme 2 (ACE2) (Hoffmann et al., 2020; Letko et al., 2020; Wan et al., 2020; Zhou et al., 2020). Host genetics has long been known to play a key function in establishing the outcome of host-pathogen interactions (susceptibility or protection) and subsequently influencing infection outcomes (mild, moderate, or severe) (De Silva and Stumpf, 2004; Samson et al., 1996; Martinson et al., 1997). Such genetic factors also contribute to population-specific traits, potentially explaining disparities in disease burden and severity across different populations, as highlighted in the context of COVID-19.

Researchers have suggested that genetic changes including single nucleotide polymorphisms (SNPs) within ACE2 could either alter the binding affinity to the virus or gene expression levels, thereby influencing an individual’s susceptibility or protection from infection (Martínez-Gómez et al., 2022; Möhlendick et al., 2021; Mahmood et al., 2022; Liu et al., 2016). While there is ample research exploring the impact of host genetics, particularly, ACE2 SNPs on SARS-CoV-2 infection in Europe, Asia, and other global regions, there is considerable knowledge gap regarding these genetic associations within the African population. This is due to the absence of a well-established and robust laboratory technology pipeline such as high throughput next-generation sequencing and genotyping approaches, which are essential to conducting similar studies (Petersen et al., 2022). Additionally, the distinct genetic diversity of Africa, shaped by early human origins (Stringer, 2016), endemic diseases (Adimulam et al., 2023), and a large socio-cultural structure (Schlebusch et al., 2012), distinguishes it from other populations (Campbell and Tishkoff, 2008; Yu et al., 2002). Consequently, the current global evidence derived from existing studies lacks comprehensive representation of genetic diversity, limiting our ability to draw consistent conclusions across populations. In sub-Saharan Africa, several SNPs have been associated with infectious diseases. For example, constant exposure to the parasite in malaria-endemic regions have driven the natural selection for protective genetic variations (Dhangadamajhi et al., 2010). The unique genetic background raises critical concerns about the generalizability of genetic findings across diverse global populations, including African populations.

ACE2-rs2285666 (C>T) and ACE2-rs2106809 (A>G) have been recognized as important SNPs that regulates ACE2 expression levels, which may, in turn affect the number of available receptor sites for SARS-CoV-2 virus attachment to cause an infection, thereby potentially conferring resistance to infection (Dhangadamajhi et al., 2010; De et al., 2021).

Ghana reflects the broader trends of Sub-Saharan Africa, with significant genetic diversity, unique epidemiological profile, and a history of malaria endemicity (WHO, 2023b). Investigating ACE2 SNPs in the Ghanaian population may reveal insights into COVID-19 susceptibility patterns and inform therapeutic strategies for future (coronavirus) outbreaks.

Investigating the association between ACE2 SNPs and the susceptibility to SARS-CoV-2 infection using SARS-CoV-2 IgG seropositivity, determined through robust methods as a proxy allows for a more inclusive and practical solution, particularly in resource-limited settings like Ghana. Other diagnostic methods, such as PCR testing, though the reference standard, are expensive and logistically difficult to scale, leading to testing strategies that primarily target specific groups rather than the broader population in such settings (COVID-19, 2024). While PCR testing detects acute infections, seropositivity provides evidence of past infections and thus captures the cumulative exposure to SARS-CoV-2 over time. This broader temporal coverage makes seropositivity particularly suitable for studying genetic susceptibility to infection in population-based studies.

By leveraging SARS-CoV-2 IgG serostatus from a highly representative population sample, this approach provides an alternative framework for conducting genetic association studies. Unlike traditional case-control studies, which require carefully matched controls and active cases, this alternative framework addresses the challenges of control selection–particularly in situations with high rates of asymptomatic cases, as seen with COVID-19 (Zhao et al., 2020; Oran and Topol, 2020). Based on this framework, this study hypothesizes that ACE2 SNPs are associated with SARS-CoV-2 infection susceptibility in the Ghanaian population. Specifically, it aims to (a) assess the sex-specific genetic association of ACE2-rs2285666 (C>T) and ACE2-rs2106809 (A>G) with SARS-CoV-2 IgG seropositivity, and (b) evaluate their genotypic and allelic frequencies.

## Materials and methods

### Study design and population

The study was a sub-study of a larger SARS-CoV-2 IgG seroprevalence study in Ghana, conducted in accordance with the previously published SeroCoV protocol (Lorenz et al., 2021). This protocol aligned with the World Health Organization’s standardized guidelines for seroepidemiological studies (WHO, 2020a).

Briefly, a non-random subset of participants was drawn from a two-stage cluster population-based household cross-sectional study where participants were recruited from three major cities–namely, Kumasi (Struck et al., 2022), Accra, and Tamale–covering the period from February 2021, and February 2022. The survey comprised three phases: the first phase between February 2021, and March 2021, in Kumasi, the second phase between June 2021, and October 2021, in Accra, and the third phase between November 2021, and February 2022, in Tamale.

The study included individuals aged 10 years and above who resided in households within selected sampling frame, provided informed consent for genetic analysis, and had their SARS-CoV-2 IgG seropositivity results, and complete demographic and SNP genotyping data available from the main study. To ensure that seropositivity reflected natural SARS-CoV-2 infection rather than vaccine-induced immunity, individuals who had received at least one of any of the rolled-out COVID-19 vaccination at the time of the main study were excluded.

### Detection of SARS-CoV-2-specific IgG seropositivity

SARS-CoV-2-specific IgG antibodies were measured in participant plasma samples using a sensitive and specific qualitative enzymed-linked immunosorbent assay (ELISA), following manufacturer’s guidelines.

Fcγ-receptor-based anti-SARS-CoV-2-specific IgG ELISA kit was developed by the Diagnostics Development Laboratory (DDL) at the Bernhard Nocht Institute for Tropical Medicine (BNITM), Hamburg, Germany (patent EP2492689) (Schmitz et al., 2011), and used to determine SARS-CoV-2 serostatus in the presented study. This ELISA operates by capturing antigen-antibody complexes via a solid-phase-bound Fcγ-receptor (FcγR), with a truncated SARS-CoV-2 nucleocapsid protein (NCP) as the target antigen and has proven to be highly specific. The assay was tested and validated using a serum panel derived from 35 COVID-19 patients with 213 longitudinal samples, as well as a control panel of negative samples containing 790 preCOVID-19 samples sourced from a variety of geographical regions, including Europe (Germany), Asia (Laos), and Africa (Ghana, Madagascar, and Nigeria), designed to address the issue of limited specificity of commercialized ELISA assays among samples originating from malaria-endemic countries (Deschermeier et al., 2022; Emmerich et al., 2021). Results were interpreted based on criteria set by the manufacturer, according to the batch specific certificate of analysis for the positive and negative controls used in the assay.

### Genomic DNA extraction

Following blood collection during the initial study, samples were centrifuged, and the cell pellet resuspended in 8M Urea for stable storage at room temperature. Genomic DNA was extracted using the Mag Maxi DNA extraction kit (Biosearch technologies, United Kingdom), according to the manufacturer’s instructions. Briefly, equal volumes of sample and lysis buffer were mixed and incubated overnight at 55°C with 20 µL protease. Magnetic particles were added, and bound DNA was washed, eluted, and measured using NanoDrop^®^.

### Primer design and information

The Modified Tetra-Primer Assay (MTPA) PCR primer design tool was used to design primers (Tanha et al., 2015) using GenBank sequence of ACE2 rs2285666 and ACE2 rs2106809 (NC_000023.10:g.15610348C>T, and NC_000023.11:g.15599938A>G), respectively.

For each SNP, the MTPA PCR primer design tool revealed two sets of primers (two outer and two inner primers) (Tanha et al., 2015; Baris et al., 2013; Etlik et al., 2008). Two distinct primer sets are employed to amplify two smaller and allele-specific fragments. The outer primers amplify the common, much larger DNA fragment containing the SNP, whereas the inner primers (allele-specific primers) amplify the two allele-specific fragments - wild-type and mutant-type allele-specific fragments - concurrently. The mutant allele-specific fragment is amplified by the forward inner primer and the reverse outer primer, while the wildtype allele-specific fragment is amplified by the reverse inner primer and the forward inner primer.

The NCBI online Primer Blast program at **(** https://www.ncbi.nlm.nih.gov/tools/primer-blast/) and the online Oligo-Analyzer 3.1 tool by Integrated DNA Technologies (IDT) **(** https://eu.idtdna.com/calc/analyzer **)** was employed to assess the specificity of the selected primer. The primers used are shown in Supplementary Table S1.

### Genotyping of ACE2 rs2285666 and rs2106809

Genotyping was performed using real-time PCR on the LineGene 9600 thermocycler (Hangzhou Bioer Technology Co. Ltd, China)**.** For each ACE2 SNP, two separate allele-specific reactions were prepared to target the mutant and wildtype alleles with concentrations, as specified in Supplementary Table S1.

PCR amplification conditions were set up as follows: initial denaturation at 95°C for 5 min. For rs2285666, amplification continued with 40 cycles of 95°C for 10 s (denaturation), 50.9°C for 20 s (annealing), and 72°C for 30 s (extension). For rs2106809, 30 cycles were performed with an annealing temperature of 55°C. After amplification, allele-specific fragment product melting curves were analysed by first heating the samples to 95°C for 15 s, cooling to 65°C for 1 min, and then gradually increasing the temperature to 97°C at a rate of 0.2°C/min for 15 s, while continuously measuring the change in fluorescence. The thermocycler calculated the negative derivative of the fluorescence change and generated a melting curve for each sample. Melting peaks were visualized as the negative derivative of fluorescence versus temperature (-dF/dT) of the melting curve for amplification products.

In each PCR run, specific known genotyped control samples were used: NA18499 (CT) and NA19118 (CC) for rs2285666, and HG02769 (AG) and NA19118 (AA) for rs2106809. Control samples were selected from the National Human Genome Research Institute (NHGRI) repository, using genotype data available through the Ensembl database (https://www.ensembl.org/), and were obtained from the Coriell Institute for Medical Research (http://www.coriell.org/). These controls were essential for validating primer specificity and optimizing PCR conditions, enabling precise differentiation between heterozygous and homozygous genotypes. For details on control samples used, see Supplementary Table S2. As an additional verification step, PCR-specific products were run on 1.5% agarose gel electrophoresis to confirm the presence and band sizes of products obtained (Supplementary Figure S1).

### Statistical analysis

Data analyses were carried out with the Statistical Package for the Social Sciences (SPSS) version 26.0 (IBM Corp. Released 2019. IBM SPSS Statistics for windows, Version 26.0. Armonk, N.Y: IBM Corp) and R studio software (R: The R Project for Statistical Computing).

The Chi-square test was used to assess Hardy-Weinberg Equilibrium (HWE) analysis for the two SNPs and compare the genotype and allele frequencies of rs2285666 (C>T) and rs2106809 (A>G) between SARSCoV-2 IgG-seropositive and seronegative participants. SARS-CoV-2 IgG seropositivity was used as a proxy to assess the susceptibility to SARS-CoV-2 infection. To determine the association between ACE2 SNPs and infection susceptibility, logistic regression was used to estimate odds ratios (ORs) and 95% confidence intervals (CIs). This analysis considered each SNP as a predictive variable while adjusting for potential confounders, including participant age, underlying health conditions, and smoking status. Additionally, the study site was included as an offset variable to account for potential variations in seropositivity due to differences in sampling times across the three study sites.

Given the ACE2 gene is located on the human X chromosome (gene locus Xp22.2), its inheritance patterns differ between sexes—males (XY) having a single copy (hemizygous) and females (XX) having two copies (homozygous or heterozygous). Therefore, separate analyses were performed to account for potential sex-specific differences. Various inheritance models, including dominant, heterozygous advantage, recessive, and allele models, were considered in the association analysis for each SNP. For all tests, the statistical significance level was set at α = 0.05. All P-values were adjusted for multiple testing using the Benjamini–Hochberg (BH) procedure, where appropriate.

## Results

### Socio-demographic and clinical characteristics of study participants

1,993 participants from 910 households in Kumasi, Accra, and Tamale were eligible for analysis. However, 354 subjects had received at least one dose of the COVID-19 vaccination and 308 did not have complete SNP genotyping data. As a result, data from 1,334 participants and 691 households were included in this analysis, with the majority (64.3%) being female subjects. To determine the relationship between ACE2 SNPs and the susceptibility to SARS-CoV-2 infection, two groups were compared: 55.6% (742) SARS-CoV-2 IgG seropositive, and 44.4% (592) SARS-CoV-2 IgG seronegative.

The median age was 33 (IQR = 24–46) years among SARS-CoV-2-IgG seronegative and 31 (IQR = 23–45) for SARS-CoV-2-IgG seropositive individuals. SARS-CoV-2-IgG seropositive and SARS-CoV-2-IgG seronegative participants varied considerably across study sites (except for Accra), demographic groups, and socio-behavioral factors (Table 1). Overall, females predominated (64.3%). Males demonstrated a seropositivity rate of 58.4%, (95%CI: 52.6%–64.2%) while females reported a seropositivity rate of (54.1%, 95%CI: 49.54%–58.61%), when seropositivity was analysed within sex. Education levels differed across participants, with primary education being the most frequently reported, and a higher proportion of seropositive individuals having no formal education compared to seronegatives. Nearly 41.5% were engaged in high-risk occupations, while the vast majority had no underlying health conditions (87.2%) and did not smoke (97.3%). Clinically, 42.7% reported no symptoms in the 12 months before the study, while 26.8% were paucisymptomatic and 30.4% symptomatic. Reported exposure to confirmed COVID-19 cases was low (5.5%), though room sharing (80.5%) and outdoor work (43.3%) were common. Most participants had 10 to fewer than 50 daily contacts, and 41.0% reported a history of travel. The full version of the table, including 95% confidence intervals is available in the Supplementary Table S3.

**TABLE 1:** Socio-demographic and clinical characteristics of study participants.

Characteristic	SARS-CoV-2-IgG seropositive N = 742	SARS-CoV-2-IgG seronegative N = 592	Overall (N = 1334)
Study Site n (%)			
Kumasi	150 (20.2%)	221 (37.3%)	371 (27.8%)
Accra	231 (31.1%)	250 (42.2%)	481 (36.1%)
Tamale	361 (48.7%)	121 (20.4%)	482 (36.1%)
**Sex n (%)**			
Female	464 (62.5%)	394 (66.6%)	858 (64.3%)
Male	278 (37.5%)	198 (33.4%)	476 (35.7%)
Age (in years)/sex strata n (%)			
10–19, Female	54 (7.3%)	42 (7.1%)	96 (7.2%)
10–19, Male	51 (6.9%)	33 (5.6%)	84 (6.3%)
20–44, Female	291 (39.2%)	246 (41.5%)	537 (40.3%)
20–44, Male	160 (21.6%)	98 (16.6%)	258 (19.3%)
≥45, Female	119 (16.0%)	106 (17.9%)	225 (16.9%)
≥45, Male	67 (9.0%)	67 (11.3%)	134 (10.0%)
Educational level n (%)			
None	208 (28.0%)	97 (16.4%)	305 (22.9%)
Primary	299 (40.3%)	263 (44.4%)	562 (42.1%)
Secondary	149 (20.1%)	141 (23.8%)	290 (21.7%)
Tertiary	86 (11.6%)	91 (15.4%)	177 (13.3%)
Primary occupation sector n (%)			
Low Risk^a^	158 (21.3%)	139 (23.5%)	297 (22.3%)
Moderate Risk^b^	140 (18.9%)	82 (13.9%)	222 (16.6%)
High Risk^c^	309 (41.6%)	244 (41.1%)	553 (41.5%)
Unknown Risk^d^	135 (18.2%)	127 (21.5%)	262 (19.6%)
Underlying conditions n (%)			
No	653 (88.0%)	510 (86.1%)	1163 (87.2%)
Yes^e^	89 (12.0%)	82 (13.9%)	171 (12.8%)
Smoking n (%)			
No	724 (97.6%)	574 (97.0%)	1298 (97.3%)
Yes	18 (2.4%)	18 (3.0%)	36 (2.7%)
Symptom profile ≤ 12 months before study recruitment n (%)			
Asymptomatic^f^	332 (44.7%)	238 (40.2%)	570 (42.7%)
Paucisymptomatic^g^	194 (26.1%)	164 (27.7%)	358 (26.8%)
Symptomatic^h^	216 (29.1%)	190 (32.1%)	406 (30.4%)
Contact with confirmed COVID-19 case n (%)			
No	716 (96.5%)	544 (91.9%)	1260 (94.5%)
Yes	26 (3.5%)	48 (8.1%)	74 (5.5%)
Shared room n (%)			
Not reported	2 (0.3%)	7 (1.2%)	9 (0.7%)
No	129 (17.4%)	122 (20.6%)	251 (18.8%)
Yes	611 (82.3%)	463 (78.2%)	1074 (80.5%)
Working time n (%)			
Not reported	250 (33.7%)	206 (34.8%)	456 (34.2%)
Both	57 (7.7%)	67 (11.3%)	124 (9.3%)
Indoor	85 (11.5%)	91 (15.4%)	176 (13.2%)
Outdoor	350 (47.1%)	228 (38.5%)	578 (43.3%)
Contact in a day n (%)			
Not reported	29 (3.9%)	27 (4.6%)	56 (4.2%)
less than 5	66 (8.9%)	89 (15.0%)	155 (11.6%)
5 to less than 10	197 (26.5%)	127 (21.5%)	324 (24.3%)
10 to less than 50	270 (36.4%)	195 (32.9%)	465 (34.9%)
50 or more	180 (24.3%)	154 (26.0%)	334 (25.0%)
Travel history n (%)			
Not reported	1 (0.1%)	4 (0.7%)	5 (0.4%)
No	455 (61.4%)	327 (55.2%)	782 (58.6%)
Yes	286 (38.5%)	261 (44.1%)	547 (41.0%)

^a^ Low risk include unemployed

^b^ Moderate risk include agriculture, building and construction, education, and civil/public servants

^c^ High risk include business/trade/retail, healthcare, and transportation

^d^ Unknown risk include not reported and other

^e^ Underlying conditions include diabetes, hypertension, heart disease, obesity, and lung disease

^f^ Asymptomatic: no symptoms

^g^ Paucisymptomatic: one or two mild symptoms reported, excluding loss of taste or smell and shortness of breath

^h^ Symptomatic: multiple or major symptoms reported including fever, loss of taste or smell, or shortness of breath.

### Genotype and allele frequencies of ACE2 SNPs

Table 2 shows the genotype and allele frequency distribution of ACE2 SNPs among the study population. Males (XY) were represented by allele frequencies due to their hemizygosity, while females (XX) were represented by genotype frequency (homozygous or heterozygous). The distribution for both SNPs were consistent with Hardy-Weinberg equilibrium, with (P = 0.697) for rs2285666 and (P = 0.366) for rs2106809.

**TABLE 2:** Genotype and allele frequency distributions among study participants.

SNP	SARS-CoV-2 IgG seropositive			SARS-CoV-2 IgG seronegative			P-value (BH-adjusted)	Overall		
		Allele Frequency			Allele Frequency				Allele Frequency	
rs2285666									C	T
	N (%)	C	T	N (%)	C	T		N (%)	0.91	0.09
Females		0.88	0.12		0.86	0.14	1.000			
CC	350 (75.4%)			285 (72.3%)			1.000	635 (47.6%)		
CT	113 (24.4%)			107 (27.2%)			1.000	220 (16.5%)		
TT	1 (0.2%)			2 (0.5%)			1.000	3 (0.2%)		
Males		0.97	0.03		0.97	0.03	1.000			
C	270 (97.1%)			193 (97.5%)			1.000	463 (34.7%)		
T	8 (2.9%)			5 (2.5%)			1.000	13 (1.0%)		
rs2106809										
									A	G
	N (%)	A	G	N (%)	A	G		N (%)	0.94	0.06
Females		0.91	0.09		0.91	0.09	1.000			
AA	386 (83.2%)			327 (83.0%)			1.000	713 (53.4%)		
AG	77 (16.6%)			67 (17.0%)			1.000	144 (10.8%)		
GG	1 (0.2%)			0 (0.0%)			1.000	1 (0.1%)		
Males		0.97	0.03		0.99	0.01	1.000			
A	270 (97.1%)			197 (99.5%)			0.414	467 (35.0%)		
G	8 (2.9%)			1 (0.5%)			0.414	9 (0.7%)		

The analysis did not reveal significant differences in overall allele or genotype frequencies between SARS-CoV-2 IgG seropositive and seronegative individuals, for both females and males. For ACE2 rs2285666, genotype distribution was 75.4% CC, 24.4% CT, and 0.2% TT in SARS-CoV-2-IgG seropositive females and 97.1% C and 2.9% T in SARS-CoV-2-IgG seropositive males, with a minor allele frequency (MAF) of 0.12 and 0.03 for the T-allele in females and males, respectively. The genotypes for ACE2 rs2106809 in SARS-CoV-2 IgG seropositive individuals were distributed as follows: 83.2% AA, 16.6% AG, 0.2% GG in females and 97.1% A and 2.9% in males. The minor allele frequency (MAF) for the G allele was found to be 0.09 and 0.03 in SARS-CoV-2-IgG seropositive females and males, respectively.

### Association between ACE2 SNPs and the susceptibility to SARS-CoV-2 infection

The association between ACE2 SNPs and the susceptibility to SARS-CoV-2 infection is shown in Table 3. To account for sex-specific differences, association analysis was conducted separately for males and females. Briefly, there was no evidence for an association between ACE2 - rs2285666 and rs2106809 SNPs and the susceptibility to SARS-CoV-2 infection under the crude model. After adjusting for age, underlying conditions, and smoking, with the study site set as an offset variable to account for study site-specific variations the analysis revealed sex-specific patterns for ACE2 rs2285666 and the susceptibility to SARS-CoV-2 infection. Among females, those with at least one copy of the T allele showed 38% reduced odds of susceptibility to SARS-CoV-2 infection under both the dominant (aOR = 0.62; 95%CI = 0.45–0.85, BH-adjusted P = 0.017) and heterozygous advantage models (aOR = 0.62; 95%CI = 0.45–0.86, BH-adjusted P = 0.017). The effect estimate under the recessive model was similar in direction but had substantially wider confidence intervals (aOR = 0.41; 95%CI = 0.03–5.22, BH-adjusted P = 0.637), reflecting the small number of individuals with two copies of the T allele. Among males, no clear direction of associations was found under the dominant (aOR = 1.00; 95% CI = 0.30–3.32; BH-adjusted P = 0.998), and recessive models (aOR = 1.00; 95% CI = 0.30–3.32; BH-adjusted P = 0.998). Notably, in the allelic model, where the C allele was treated as the effect allele, an increased odds of susceptibility to SARS-CoV-2 infection was observed (aOR = 1.62, BH-adjusted P = 0.017), which aligns with the protective role associated with the T allele observed under the dominant and heterozygous models. These findings may be explained by a potential sex-specific genetic effect, with the T allele of ACE2 rs2285666 being more protective against SARS-CoV-2 infection in females than males (Table 3).

**TABLE 3:** Association between rs2285666 and rs2106809 and susceptibility to SARS-COV-2 infection.

SNP	Genotype	cOR (95%CI)	P-value	aOR (95%CI)	P-value (BH-adjusted)
rs2285666 (C>T)					
Dominant					
Females	CC vs CT + TT	0.85 (0.63–1.16)	0.303	0.62 (0.45–0.85)	**0.017**
Males	C vs T	1.14 (0.37–3.55)	0.816	1.00 (0.30–3.32)	0.998
Heterozygous Advantage					
Females	CT vs CC + TT	0.86 (0.64–1.17)	0.349	0.62 (0.45–0.86)	**0.017**
Recessive					
Females	TT vs CC + CT	0.42 (0.04–4.69)	0.483	0.41 (0.03–5.22)	0.637
Males	T vs C	1.14 (0.37–3.55)	0.816	1.00 (0.30–3.32)	0.998
Allelic					
Females	T vs C	1.17 (0.87–1.59)	0.303	1.62 (1.17–2.25)	**0.017**
Males	T vs C	0.87 (0.28–2.71)	0.816	1.00 (0.30–3.31)	0.998
rs2106809 (A>G)					
Dominant					
Females	AA vs AG + GG	0.99 (0.69–1.41)	0.940	0.72 (0.49–1.05)	0.195
Males	A vs G	5.84 (0.72–47.04)	0.098	4.77 (0.50–45.47)	0.253
Heterozygous Advantage					
Females	AG vs AA + GG	0.97 (0.68–1.39)	0.873	0.71 (0.49–1.04)	0.195
Recessive					
Females	GG vs AA + AG	NA^a^	NA^a^	NA^a^	NA^a^
Males	G vs A	5.84 (0.72–47.04)	0.098	4.77 (0.50–45.47)	0.253
Allelic					
Females	G vs A	1.01 (0.71–1.45)	0.940	1.39 (0.95–2.03)	0.195
Males	G vs A	0.17 (0.02–1.38)	0.098	0.21 (0.02–2.00)	0.253

^a^ Odds ratio could not be computed due to small sample size of females with GG, genotype. Bolded p-values indicate statistically significant associations (p<0.05).

## Discussion

The COVID-19 pandemic presented with varying infection rates between and within populations (Bialek et al., 2020; WHO, 2023a). Factors contributing to this disparity, includes behavioral (Betsch et al., 2020; Chinazzi et al., 2020; Courtemanche et al., 2020), social (Dorn et al., 2020; Lewer et al., 2020; Yancy, 2020), physiological (Kellam and Barclay, 2020; Guan et al., 2020), and biological variables (Kellam and Barclay, 2020; Eshetie et al., 2023). As of June 26, 2022, there were 542,018,955 confirmed cases globally, with WHO regional disparities in distribution (WHO, 2023): 1.7% in the African region, 29.9% in the Americas, 4.0% in the Eastern Mediterranean, 41.8% in Europe, 10.8% in South-East Asia, and 11.7% in the Western Pacific. In Ghana, over 100,000 confirmed cases and 1,450 deaths were recorded during the same period, reflecting the pandemic’s varied impact across regions and populations (GHS, 2023).

ACE2 is the major receptor for SARS-CoV-2, which mediates cell entry (WHO, 2023a). Within the complex network of disease pathogenesis, SNPs in key genes have long been recognized as critical influencers affecting an individual’s susceptibility to infection (Gray et al., 2000). ACE2 SNPs have already been linked to various human illnesses, including hypertension (Bosso et al., 2020), heart failure (Chaoxin et al., 2013), malaria (Dhangadamajhi et al., 2010), and diabetes (Chaoxin et al., 2013). Recent findings have shown inconsistent findings on the association between ACE2 SNPs and the likelihood of SARS-CoV-2 infection, with an emphasis on Eurasian populations and limited investigations undertaken in African populations (Petersen et al., 2022). Differences in these findings may be attributed to genetic and environmental factors within the investigated populations (Price et al., 2010; Aschard et al., 2010), as well as variations in study design and methodology used (Visscher et al., 2017).

The two x-chromosomal ACE2 mutations rs2285666 (C>T) and rs2106809 (A>G) investigated in this study have been associated with elevated levels of angiotensin II (AngII), which is known to protect against severe malaria (Dhangadamajhi et al., 2010). Because AngII levels are regulated by ACE2 activity within the renin-angiotensin-aldosterone system (RAAS) (Ferrario, 2006), this indicates a decrease in ACE2 expression levels, also associated with these polymorphisms (Dhangadamajhi et al., 2010; Chen et al., 2018). However, analysis of publicly available GTEx data indicates that both rs2285666 and rs2106809 are associated with increased ACE2 expression in multiple tissues, particularly in the hypothalamus, nucleus accumbens, and pituitary, suggesting a more complex and tissue-specific regulatory effect than previously assumed (GTEx Portal, 2025). Membrane-bound (mACE2) and circulating or soluble ACE2 (cACE2 or sACE2), have been reported to retain catalytic activity and contain SARS-CoV-2 binding sites, which may contribute to onset of disease or disease progression (Medina-Enríquez et al., 2020; Yeung et al., 2021). Understanding how these polymorphisms modulate ACE2 expression across different tissues, particularly in populations with varied genetic backgrounds is needed.

The overall genotype and allele frequency distribution were comparable between SARS-CoV-2-IgG seropositive and seronegative groups for both females and males. However, ACE2-rs2285666 (T-allele) was found to be associated with protection from SARS-CoV-2 infection among the female population. This association was observed after adjusting for age, underlying medical conditions, participants’ smoking status, and study site sampling variations. In males, no discernible association was observed. This finding may be explained by a sex-specific functional role of the T-allele. Dhangadamajhi et al., (2010) observed that the T allele reduces ACE2 expression levels in women, specifically (Dhangadamajhi et al., 2010). Factors, such as sex hormones (Lott et al., 2023) and diverse immune responses (Ghosh and Klein, 2017), may contribute to such an association. Future studies investigating the interplay between sex hormones, humoral responses, and genetics would help understanding sex-specific differences in susceptibility to SARS-CoV-2 infection. Consistent with the findings of the rs2285666 SNP in this study, Möhlendick et al., (2021) reported an almost twofold increased SARS-CoV-2 infection susceptibility among individuals carrying the GG genotype (OR = 1.91, 95%CI: 1.13–3.24; P = 0.02) or G allele (OR = 1.88, 95%CI: 1.12–3.16; P = 0.02) of the rs2285666 SNP. However, differences exist as the previous study employed a highly selected cohort of PCR-confirmed SARS-COV-2 cases and controls, while the current study used population-based SARS-CoV-2-specific IgG serosurvey data. In addition, the previous study did not consider possible sex-specific ACE2 SNP-associated SARS-CoV-2 susceptibility differences as observed in this study. The frequency of the T allele varies across populations, with a higher prevalence observed in Asian populations compared to European and African populations (Srivastava et al., 2020). This suggests that genetic susceptibility to SARS-CoV-2 infection may differ among ethnic groups. Although studies have reported higher SARS-CoV-2 infection rates in males than females (Do Nascimento et al., 2020; The COVID-19 Sex-Disaggregated Data Tracker, 2025; Conti and Younes, 2020; Rostami et al., 2021; Vahidy et al., 2021), the findings of this study should be interpreted within the broader context of multiple factors influencing infection susceptibility.

Regarding the ACE2 rs2106809 (A>G), there was no significant association with SARS-CoV-2 infection susceptibility in both sexes. Although this finding does not necessarily negate the importance of the rs2106809 SNP in SARS-CoV-2 infection, they underscore how factors such as age, underlying condition, and smoking shape an individual’s susceptibility. This accentuates the necessity for comprehensive and multifactorial methods in genetic studies. The lack of a significant association could be attributed to the unique genetic makeup of the Ghanaian population, suggesting that different populations may yield diverse results, underscoring the need for replicative studies. While other studies have explored how this SNP is associated with the severity of COVID-19 (Cafiero et al., 2021; Karakaş Çelik et al., 2021; Mohammadi-Berenjestanaki et al., 2023; Sabater Molina et al., 2022)**,** this study specifically explored its predisposition to SARS-CoV-2 infection, with insufficient evidence available for making direct comparisons.

It is worth noting that several other critical genes have been implicated in COVID-19 genetic studies, in addition to ACE2, across different populations. Numerous loci have shown associations with both susceptibility to infection and disease severity. Early genome-wide association study (GWAS) highlighted the significance of genes such as SLC6A20, LZTFL1, CCR9, FYCO1, CXCR6, and XCR1 as key determinants of SARS-CoV-2 susceptibility (lead variant rs2271616) (Niemi et al., 2021). Furthermore, IFNAR2 and IL10RB have been linked to increased SARS-CoV-2 infection susceptibility (Kasela et al., 2021). Additional studies have further reported associations with the genes ABO, DPP9, HLA, OAS1, SLC22A31, SFTPD, AND CXCR6 (Ellinghaus et al., 2020; Pairo-Castineira et al., 2020; Pairo-Castineira et al., 2023). Integrating polygenic risk scores and fine-mapping approaches in COVID-19 genetic studies could help clarify the role of these loci in diverse genetic backgrounds.

This study benefited from a population-representative sample derived from a household-based cross-sectional survey and utilized a highly specific ELISA assay for estimating seroprevalence. This study also finds its strength by the use of a robust genotyping assay bolstered by the inclusion of already known genotyped samples as controls, improving its sensitivity and specificity for accurate and reliable SNP detection. However, the study has several limitations. While it aimed for a population-representative sample, the specific eligibility criteria set out for this study reduced its initial representativeness. The exclusion of vaccinated individuals may have selectively removed certain groups from the analyses; thus, findings should be interpreted cautiously. The relatively small sample size for males may have limited the ability to detect significant associations in this study. Additionally, the cross-sectional design of the main study only allows for the assessment of associations at a single point in time, lacking the ability to account for the temporal dynamics of the COVID-19 epidemic. Another limitation is the inability to draw firm conclusions about the functional consequences of the investigated ACE2 SNPs on ACE2 expression, which precludes the direct assessment of the mechanistic link between SARS-CoV-2 infection susceptibility. Furthermore, antibody waning may have led to the misclassification of some previously infected individuals as seronegative.

The extended time frame of sample collection may have introduced heterogeneity due to the emergence of SARS-CoV-2 variants which may have affected antibody detection. However, this variability was more pronounced in the SARS-CoV-2 Spike (S) protein. In our study, serological detection was based on antibodies to the NCP, which is more conserved across variants, thereby minimizing potential bias related to antigenic variation. The study also did not quantify SARS-CoV-2 IgG antibody levels in participant plasma. Additionally, using seropositivity as a proxy for infection may exclude individuals who succumbed to COVID-19 prior to serological sampling, potentially introducing bias toward non-severe cases. However, the primary aim of this study was to assess genetic associations with susceptibility to SARS-CoV-2 infection, rather than disease severity. Future research that includes hospitalized patients with severe disease could provide further insights into the role of ACE2 polymorphisms in COVID-19 severity, thereby enhancing our understanding of the genetic basis of the disease in Africa.

Recent advancements integrating COVID-19 GWAS with single-cell RNA sequencing (scRNA-seq) data have provided deeper insights into the immune cell types and critical genes associated with SARS-CoV-2 infection. Tools such as scPagwas (Ma et al., 2023) and scDRS (Zhang et al., 2022) have proven powerful in identifying immune pathways involved in the disease pathogenesis. These methodologies offer significant potential for investigating COVID-19 in populations like the Ghanaian cohort studied in this research. Future studies applying this approach could help further elucidate the complex immune interactions and gene-environment factors that influence COVID-19 susceptibility and disease outcomes in African populations.

## Conclusion

The study concludes that ACE2 rs228566 but not rs2106809 is associated with an individual’s susceptibility to SARS-CoV-2 infection among the Ghanaian female population. The observed sex-specific association for the ACE2 rs2285666 T allele highlights the importance of considering host genetics and immunological differences in understanding SARS-CoV-2 vulnerability. While further research is needed to clarify the underlying mechanisms and to validate these findings across diverse populations, the results underscore the potential for incorporating genetic markers into clinical risk stratification models and public health planning. In resource-limited settings like Ghana, integrating host genetic data into national pandemic preparedness plans could enhance resource management by prioritizing vaccination deployments to vulnerable populations. This in turn could facilitate tailored public health responses through enhanced preventive measures, specialized care pathways, or design of sentinel surveillance systems for susceptible individuals. These findings support the expansion of population-based genomic surveillance to enable precision public health responses in low resource settings and beyond.

## Data Availability

The original contributions presented in the study are included in the article/Supplementary Material, further inquiries can be directed to the corresponding authors.
