# Supplementary material for: Genetic association of ACE2 rs2285666 (C>T) and rs2106809 (A>G) and susceptibility to SARS-CoV-2 infection among the Ghanaian population
Source: Front Genet. 2025 May 26;16:1555515. doi: 10.3389/fgene.2025.1555515 (PMC12146278; doi:10.3389/fgene.2025.1555515)
Supplement: Supplementary file 1 [file Supplementaryfile1.docx]

**Supplementary Figure 1: Allele-Specific Oligonucleotide PCR and Melting Curve analysis for SNP Genotyping**


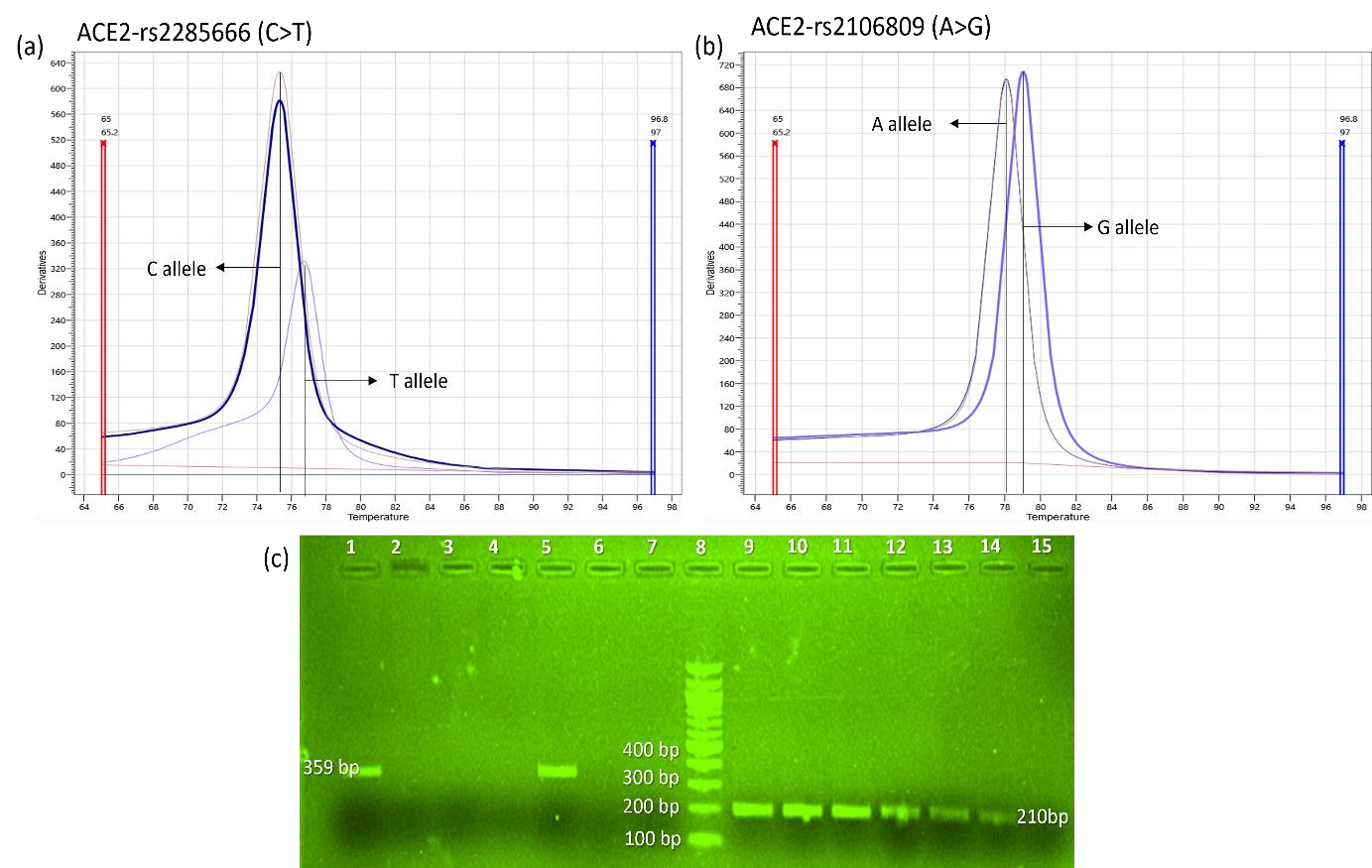


Figure 1 ACE2-rs2106809 (A>G) and ACE2-rs2285666 (C>T) genotyping by real-time ASO-PCR with Melting Curve Analysis (MCA)

**(a)** Melting curves for mutant-T and wildtype-C allele-specific products of ACE2-rs2285666 (C>T) for the control samples: NA19118 (CC) and NA18479 (CT); profile with a single peak at 76.5°C representing the mutant-T allele-specific product and profile with a single peak at 75.0°C representing the wildtype-C allele-specific product. The line with no peak represents NA19118 in the reaction targeting the T-allele and the no-template control (NTC) **(b)** Melting curves for mutant-G and wildtype-A allele-specific products of ACE2-rs2106809 (A>G) for the control samples: NA19118 (AA) and HG02769 (AG); profile with a single peak at 79.3°C representing the mutant-G allele-specific product and profile with a single peak at 77.7°C representing the wildtype-A allele-specific product. The line with no peak represents NA19118 in the reaction targeting the G-allele and the no-template control (NTC) **(c)** 1.5% agarose gel electrophoresis of PCR-specific products separated the distinct PCR products of sizes 359bp and 210bp for the mutant-G and wildtype-A alleles of ACE2 rs2106809 (A>G), respectively. Lanes 1-7 represent ASO-PCR real-time amplification of mutant (G) alleles, while Lanes 9-15 represent ASO-PCR real-time amplification of wildtype (A) allele. Lanes 5 and 13 contain the heterozygous HG02769 (AG) control sample. Lanes 6 and 14 contain the homozygous NA19118 (AA) control sample. Lanes 7 and 15 represent the NTC (no-template control) sample. Unknown samples are contained in Lanes 1-4 and 9-12. Lane 8 contains the DNA ladder.
