# Supplementary material for: Genetic association of ACE2 rs2285666 (C>T) and rs2106809 (A>G) and susceptibility to SARS-CoV-2 infection among the Ghanaian population
Source: Front Genet. 2025 May 26;16:1555515. doi: 10.3389/fgene.2025.1555515 (PMC12146278; doi:10.3389/fgene.2025.1555515)
Supplement: Supplementary file 2 [file Table1.docx]

**Supplementary Table 1: Primers information**

| Primer | Primer sequence (5’ – 3’) | Tm  (°C) | Ta (°C) | Product Tm (°C) | Product size |
| --- | --- | --- | --- | --- | --- |
| **ACE2 – rs2285666 (C>T)** | | | | | |
| FIP (T *allele)* | CATAATCACTACTAAAAATTAGTATCT | 51.4 | 53.8 | 76.2 | 369 |
| RIP (C *allele*) | CTTATTACTTGAACCAGGGAG | 53.7 |  | 75.3 | 326 |
| FOP | ACTACATGAACTTTAAAGAATTACAA | 53.5 |  |  | 648 |
| ROP | AGATCTGGTTATTTCAATGAC | 53.0 |  |  |  |
| **ACE2 – rs2106809 (A>G)** | |  |  |  |  |
| FIP (G *allele*) | TTTTTTTCCATATCTCTATCTGATTGG | 52.2 | 52.4 | 74.1 | 359 |
| RIP (A *allele*) | TGATGTAGAAGTGTGGAGACGT | 55.6 |  | 73.3 | 210 |
| FOP | CTCACCAAGAAATAGCTACAAAGT | 53.5 |  |  | 521 |
| ROP | GACTTGGGAATTCCTTTCATC | 51.5 |  |  |  |

***FIP- Forward inner primer; RIP- Reverse inner primer; FOP- Forward outer primer; ROP- Reverse outer primer; Tm- Melting temperature; Ta- Annealing temperature. The mismatches of the primers are emphasized in the underline.***
