# Supplementary material for: Genetic association of ACE2 rs2285666 (C>T) and rs2106809 (A>G) and susceptibility to SARS-CoV-2 infection among the Ghanaian population
Source: Front Genet. 2025 May 26;16:1555515. doi: 10.3389/fgene.2025.1555515 (PMC12146278; doi:10.3389/fgene.2025.1555515)
Supplement: Supplementary file 3 [file Table2.docx]

**Supplementary Table 2: Genotyping Control Samples Information**

| **Sample** | **Genotype** | **Population** | **SNP** |
| --- | --- | --- | --- |
| NA18499 | CT | Yoruba in Ibadan, Nigeria | rs2285666 C>T) |
| HG02769 | AG | Gambian in Western Division, The Gambia | rs2106809 (A>G) |
| NA19118 | CC/AA | Yoruba in Ibadan, Nigeria | Homozygous control sample for both SNPs |
