# Supplementary material for: Genetic association of ACE2 rs2285666 (C>T) and rs2106809 (A>G) and susceptibility to SARS-CoV-2 infection among the Ghanaian population
Source: Front Genet. 2025 May 26;16:1555515. doi: 10.3389/fgene.2025.1555515 (PMC12146278; doi:10.3389/fgene.2025.1555515)
Supplement: Supplementary file 4 [file Table3.docx]

**Supplementary Table 3: Socio-demographic and Clinical Characteristics of Study Participants**

| **Characteristic** | **SARS-CoV-2-IgG seropositive**  **N = 742** | **SARS-CoV-2-IgG seronegative**  **N = 592** | **Overall**  **(N = 1334)** |
| --- | --- | --- | --- |
| **Study Site n (%, 95%CI)** | | | |
| Kumasi | 150 (20.2%, 13.8 – 26.6) | 221 (37.3%, 30.9 – 43.7) | 371 (27.8%, 23.2 – 32.4) |
| Accra | 231 (31.1%, 25.1 – 37.1) | 250 (42.2%, 36.1 – 48.3) | 481 (36.1%, 31.8 – 40.4) |
| Tamale | 361 (48.7%, 43.5 – 53.9) | 121 (20.4%, 13.2 – 27.6) | 482 (36.1%, 31.8 – 40.4) |
| **Sex n (%)** |  |  |  |
| Female | 464 (62.5%, 58.1 – 66.9) | 394 (66.6%, 61.9 – 71.3) | 858 (64.3%, 61.1 – 67.5) |
| Male | 278 (37.5%, 31.8 – 43.2) | 198 (33.4%, 26.8 – 40.0) | 476 (35.7%, 31.4 – 40.0) |
| **Age (in years)/sex strata n (%, 95%CI)** | | | |
| 10-19, Female | 54 (7.3%, 0.4 – 14.2) | 42 (7.1%, -0.7 – 14.9) | 96 (7.2%, 2.0 – 12.4) |
| 10-19, Male | 51 (6.9%, -0.1 – 13.9) | 33 (5.6%, -2.2 – 13.4) | 84 (6.3%, 1.1 – 11.5) |
| 20-44, Female | 291 (39.2%, 33.6 – 44.8) | 246 (41.5%, 35.3 – 47.7) | 537 (40.3%, 36.2 – 44.4) |
| 20-44, Male | 160 (21.6%, 15.2 – 28.0) | 98 (16.6%, 9.2 – 24.0) | 258 (19.3%, 14.5 – 24.1) |
| ≥ 45, Female | 119 (16.0%, 9.4 – 22.6) | 106 (17.9%, 10.6 – 25.2) | 225 (16.9%, 12.0 – 21.8) |
| ≥ 45, Male | 67 (9.0%, 2.1 – 15.9) | 67 (11.3%, 3.7 – 18.9) | 134 (10.0%, 4.9 – 15.1) |
| **Educational level n (%, 95%CI)** | | | |
| None | 208 (28.0%, 21.9 – 34.1) | 97 (16.4%, 9.0 – 23.8) | 305 (22.9%, 18.2 – 27.6) |
| Primary | 299 (40.3%, 34.7 – 45.9) | 263 (44.4%, 38.4 – 50.4) | 562 (42.1%, 38.0 – 46.2) |
| Secondary | 149 (20.1%, 13.7 – 26.5) | 141 (23.8%, 16.8 – 30.8) | 290 (21.7%, 17.0 – 26.4) |
| Tertiary | 86 (11.6%, 4.8 – 18.4) | 91 (15.4%, 8.0 – 22.8) | 177 (13.3%, 8.3 – 18.3) |
| **Primary occupation sector n (%, 95%CI)** | | | |
| Low Risk^a^ | 158 (21.3%, 14.9 – 27.7) | 139 (23.5%, 16.5 – 30.5) | 297 (22.3%, 17.6 – 27.0) |
| Moderate Risk^b^ | 140 (18.9%, 12.4 – 25.4) | 82 (13.9%, 6.4 – 21.4) | 222 (16.6%, 11.7 – 21.5) |
| High Risk^c^ | 309 (41.6%, 36.1 – 47.1) | 244 (41.1%, 34.9 – 47.3) | 553 (41.5%, 37.4 – 45.6) |
| Unknown Risk^d^ | 135 (18.2%, 11.7 – 24.7) | 127 (21.5%, 14.4 – 28.6) | 262 (19.6%, 14.8 – 24.4) |
| **Underlying conditions n (%, 95%CI)** | | | |
| No | 653 (88.0%, 85.5 – 90.5) | 510 (86.1%, 83.1 – 89.1) | 1163 (87.2%, 85.3 – 89.1) |
| Yes^e^ | 89 (12.0%, 5.2 – 18.8) | 82 (13.9%, 6.4 – 21.4) | 171 (12.8%, 7.8 – 17.8) |
| **Smoking n (%)** | | | |
| No | 724 (97.6%, 96.5 – 98.7) | 574 (97.0%, 95.6 – 98.4) | 1298 (97.3%, 96.4 – 98.2) |
| Yes | 18 (2.4%, -4.7 – 9.5) | 18 (3.0%, -4.9 – 10.9) | 36 (2.7%, -2.6 – 8.0) |
| **Symptom profile ≤ 12 months before study recruitment n (%, 95%CI)** | | | |
| Asymptomatic^f^ | 332 (44.7%, 39.4 – 50.0) | 238 (40.2%, 34.0 – 46.4) | 570 (42.7%, 38.6 – 46.8) |
| Paucisymptomatic^g^ | 194 (26.1%, 19.9 – 32.3) | 164 (27.7%, 20.9 – 34.5) | 358 (26.8%, 22.2 – 31.4) |
| Symptomatic^h^ | 216 (29.1%, 23.0 – 35.2) | 190 (32.1%, 25.5 – 38.7) | 406 (30.4%, 25.9 – 34.9) |
| **Contact with confirmed COVID-19 case n (%, 95%CI)** | | | |
| No | 716 (96.5%, 95.2 – 97.8) | 544 (91.9%, 89.6 – 94.2) | 1260 (94.5%, 93.2 – 95.8) |
| Yes | 26 (3.5%, -3.6 – 10.6) | 48 (8.1%, 0.4 – 15.8) | 74 (5.5%, 0.3 – 10.7) |
| **Shared room n (%, 95%CI)** | | | |
| Not reported | 2 (0.3%, -7.3 – 7.9) | 7 (1.2%, -6.9 – 9.3) | 9 (0.7%, -4.7 – 6.1) |
| No | 129 (17.4%, 10.9 – 23.9) | 122 (20.6%, 13.4 – 27.8) | 251 (18.8%, 14.0 – 23.6) |
| Yes | 611 (82.3%, 79.3 – 85.3) | 463 (78.2%, 74.4 – 82.0) | 1074 (80.5%, 78.1 – 82.9) |
| **Working time n (%, 95%CI)** | | | |
| Not reported | 250 (33.7%, 27.8 – 39.6) | 206 (34.8%, 28.3 – 41.3) | 456 (34.2%, 29.8 – 38.6) |
| Both | 57 (7.7%, 0.8 – 14.6) | 67 (11.3%, 3.7 – 18.9) | 124 (9.3%, 4.2 – 14.4) |
| Indoor | 85 (11.5%, 4.7 – 18.3) | 91 (15.4%, 8.0 – 22.8) | 176 (13.2%, 8.2 – 18.2) |
| Outdoor | 350 (47.1%, 41.9 – 52.3) | 228 (38.5%, 32.2 – 44.8) | 578 (43.3%, 39.3 – 47.3) |
| **Contact in a day n (%, 95%CI)** | | | |
| Not reported | 29 (3.9%, -3.1 – 10.9) | 27 (4.6%, -3.3 – 12.5) | 56 (4.2%, -1.1 – 9.5) |
| less than 5 | 66 (8.9%, 2.0 – 15.8) | 89 (15.0%, 7.6 – 22.4) | 155 (11.6%, 6.6 – 16.6) |
| 5 to less than 10 | 197 (26.5%, 20.3 – 32.7) | 127 (21.5%, 14.4 – 28.6) | 324 (24.3%, 19.6 – 29.0) |
| 10 to less than 50 | 270 (36.4%, 30.7 – 42.1) | 195 (32.9%, 26.3 – 39.5) | 465 (34.9%, 30.6 – 39.2) |
| 50 or more | 180 (24.3%, 18.0 – 30.6) | 154 (26.0%, 19.1 – 32.9) | 334 (25.0%, 20.4 – 29.6) |
| **Travel history n (%, 95%CI)** | | | |
| Not reported | 1 (0.1%, -6.1 – 6.3) | 4 (0.7%, -7.5 – 8.9) | 5 (0.4%, -5.1 – 5.9) |
| No | 455 (61.4%, 56.9 – 65.9) | 327 (55.2%, 49.8 – 60.6) | 782 (58.6%, 55.1 – 62.1) |
| Yes | 286 (38.5%, 32.9 – 44.1) | 261 (44.1%, 38.1 – 50.1) | 547 (41.0%, 36.9 – 45.1) |
